# Anti-Streptococcal Activity of β-Thujaplicin and Its Effect on Natural Low-Level Resistance of *Streptococcus* Species to Aminoglycoside Antibiotics

**DOI:** 10.3390/pathogens15090978

**Published:** 2026-09-15

**Authors:** Adriana Marcinów, Elżbieta Piątkowska, Krzysztof Kujawa

**Affiliations:** 1 Student Scientific Association of Pharmaceutical Microbiology “MICRO&Pharm”, Faculty of Pharmacy, Wroclaw Medical University, 50-556 Wroclaw, Poland; 2 Department of Pharmaceutical Microbiology and Parasitology, Faculty of Pharmacy, Wroclaw Medical University, 50-556 Wroclaw, Poland; 3 Statistical Analysis Centre, Wroclaw Medical University, 50-556 Wroclaw, Poland

**Keywords:** β-thujaplicin, hinokitiol, aminoglycoside, *Streptococcus*, synergism

## Abstract

β-thujaplicin (BTJ) is an organic compound with medicinal potential. Its activity against pathogenic *Streptococcus* species has been insufficiently investigated. This study aimed to evaluate the antimicrobial activity of β-thujaplicin against more than 30 reference and clinical isolates of *Streptococcus pyogenes*, *Streptococcus agalactiae*, and *Streptococcus pneumoniae*, and to assess its effect on the intrinsic low-level resistance of these bacteria to aminoglycosides. Susceptibility of β-thujaplicin and selected aminoglycosides was determined using the broth microdilution method. The modulatory effect of β-thujaplicin on aminoglycoside activity was assessed using the checkerboard assay, with interactions evaluated based on the fractional inhibitory concentration index (FICI). β-thujaplicin exhibited antimicrobial activity against *S. pyogenes* and *S. pneumoniae*, with minimum inhibitory concentration (MIC) values ranging from 16 to 32 µg/mL, exceeding the activity of amikacin (AN). Synergistic interactions between β-thujaplicin (2–8 µg/mL) and aminoglycosides were observed in 100% of *S. pneumoniae* strains tested, 50% of *S. pyogenes*, and 80% of *S. agalactiae* isolates. In the presence of β-thujaplicin, aminoglycoside MICs decreased by 2- to 64-fold depending on the species. β-thujaplicin demonstrates in vitro bactericidal activity against most tested Streptococcus strains and exhibits synergistic effects when combined with aminoglycosides. These findings suggest that β-thujaplicin may reduce the natural resistance of strepto-cocci to these antibiotics.

## 1. Introduction

One of the plant-derived compounds with potential antimicrobial properties is β-thujaplicin (BTJ), also known as hinokitiol [1]. It is a naturally occurring component of essential oils extracted from the heartwood of various *Cupressaceae* family members, including *Chamaecyparis taiwanensis*, *Chamaecyparis obtusa*, *Thuja plicata*, *Thuja occidentalis*, *Thujopsis dolabrata*, *Juniperus chinensis*, and *Juniperus conferta* [2,3,4]. BTJ exhibits diverse antibacterial activity against both Gram-positive and Gram-negative microorganisms [5]. In addition to its antimicrobial effects, BTJ has anti-inflammatory, antioxidant, and anticancer properties [6,7,8,9,10]. One of the significant advantages of this compound is its low tissue toxicity [5,10]. Due to these properties, β-thujaplicin was examined in vitro; the results suggest its potential application in infection prevention and cosmetology. Moreover, its use in the treatment of bacterial infections cannot be ruled out [8,11,12,13]. This is particularly relevant in the context of the growing global problem of multidrug resistance, which necessitates the search for agents with novel mechanisms of action, including inhibitors of both natural and acquired bacterial resistance to antibiotics.

In May 2024, the World Health Organization (WHO) published a list of bacterial priority pathogens of public health importance, emphasizing the urgent need for research related to prevention options, control of infections, and antimicrobial resistance [14]. For the first time, the list included three species from the genus *Streptococcus* spp.—*S. pyogenes*, *S. agalactiae*, and *S. pneumoniae*. This inclusion may be linked to the post-pandemic rise in invasive infections caused by *S. pyogenes*, as well as the gradual decline in the susceptibility of *S. pneumoniae* and *S. agalactiae* to standard antimicrobial therapies [15,16,17,18]. In addition to acquired resistance mechanisms, *Streptococcus* spp. also exhibit intrinsic resistance to certain antibiotics, such as ceftazidime, fusidic acid, and aminoglycosides [19]. In the case of aminoglycosides, this resistance is primarily due to their limited ability to penetrate bacterial cell walls. However, this natural resistance may be overcome through combination therapy with cell wall synthesis inhibitors (penicillins, glycopeptides), which act synergistically by facilitating aminoglycoside entry into the bacterial cell and reaching their target site of action [20,21]. Due to the multiple modes of BTJ antimicrobial action, it is hypothesized that this compound may also influence the anti-streptococcal activity of aminoglycosides as well [8,22,23].

This experimental study aimed to evaluate the antimicrobial activity of β-thujaplicin against clinically relevant *Streptococcus* species (*S. pneumoniae*, *S. pyogenes*, *S. agalactiae*) and to assess its potential impact on the activity of aminoglycosides, to which these bacteria are naturally less susceptible.

## 2. Materials and Methods

### 2.1. Bacterial Strains

This study employed reference strains of *Streptococcus pyogenes* ATCC 19615, *Streptococcus agalactiae* ATCC 12386, and *Streptococcus pneumoniae* ATCC 49619, as well as thirty clinical isolates of the aforementioned species, recovered between 2023 and 2024 from infections in patients hospitalized in Wroclaw, Poland. Species identification of the strains relied on phenotypic characteristics, including colony morphology, hemolysis pattern, and susceptibility to optochin (Oxoid, Basingstoke, UK) and bacitracin (Oxoid, Basingstoke, UK). Species confirmation was performed with matrix-assisted laser desorption/ionization time-of-flight mass spectrometry (MALDI-TOF MS; MBT Compass HT software, IVD reference library v.2023, Bruker Daltonics, Bremen, Germany). Details regarding the origin of the strains are presented in Table 1.

The strains were stored at −70 °C in brain–heart infusion (BHI, Becton Dickinson, Sparks, MD, USA) broth supplemented with 20% glycerol (Chemland, Katowice, Poland). Prior to testing, isolates were cultured overnight at 37 °C in a 5% CO_2_-enriched atmosphere on Columbia agar with 5% sheep blood (CA, Becton Dickinson, Sparks, MD, USA; BioAnimali, Wroclaw, Poland), pH 7.2. The reference strains *Staphylococcus aureus* ATCC 29213 and *Enterococcus faecalis* ATCC 29212 were used as quality control strains for determining the minimum inhibitory concentration (MIC) of antibiotics and were incubated under aerobic conditions.

### 2.2. Solutions of the Tested Compounds

Stock solutions of β-thujaplicin (BTJ; Sigma-Aldrich, St. Louis, MO, USA, purity 99%) were prepared at a concentration of 10 mg/mL in DMSO (Sigma-Aldrich, St. Louis, MO, USA) and stored protected from light. Antibiotic stock solutions (Sigma-Aldrich, St. Louis, MO, USA) included gentamicin (GM), amikacin (AN), tobramycin (TOB), and tetracycline (T), each dissolved in sterile distilled water at a concentration of 5 mg/mL. A 5 mg/mL erythromycin (E) solution was prepared in 95% ethanol (Stanlab, Lublin, Poland). From these stock solutions, two-fold serial dilutions of the test compounds were prepared in cation-adjusted Mueller–Hinton broth (MHB; Becton Dickinson, Sparks, MD, USA), supplemented with 5% lysed horse blood (BioAnimali, Wroclaw, Poland) and 20 mg/L β-NAD (Sigma-Aldrich, St. Louis, MO, USA) (MH-F).

### 2.3. Methods

To characterize the antibiotic susceptibility of the tested strains, the disc diffusion method was applied in accordance with the guidelines of the European Committee on Antimicrobial Susceptibility Testing (EUCAST) [24]. Based on these phenotypic screening tests, the presence of resistance to macrolides, lincosamides, and streptogramins B (MLS_B_, MS_B_) was also determined.

Susceptibility to aminoglycosides and β-thujaplicin was evaluated against MIC values, determined in duplicate using the broth microdilution method (ISO 20776-1:2019) with modifications for fastidious organisms [25]. Testing was performed in MH-F broth using 96-well microtitre plates. The concentration range assessed for both antibiotics and BTJ was 0.03–1024 µg/mL. The antimicrobial activity of the DMSO solvent used for BTJ was also examined; its concentration in the highest BTJ dilution did not exceed 12.5%, and in all remaining dilutions, it was below 6.25%. The final inoculum concentration was approximately 5 × 10^5^ CFU/mL per well. Incubation was carried out for 18 ± 2 h at 35 °C under a microaerophilic atmosphere containing 5% CO_2_. MICs were read manually and defined as the lowest concentration of the test compound at which no visible pellet of bacterial growth or hemolysis was observed. *Streptococcus pneumoniae* ATCC 49619 served as the quality control strain. Additionally, MICs of antibiotics were determined for *Staphylococcus aureus* ATCC 29213 and *Enterococcus faecalis* ATCC 29212 in cation-adjusted Mueller–Hinton broth (MHB) following EUCAST recommendations [24,26]. Due to the absence of clinical breakpoints for aminoglycosides in *Streptococcus* spp., strains were classified as wild-type and non-wild-type based on epidemiological cut-off values (ECOFFs) [27].

For both aminoglycosides and BTJ, minimum bactericidal concentration (MBC) values were also determined. Ten microlitres of bacterial suspension from each well containing an antibiotic or BTJ concentration ≥ ½ MIC were plated onto Columbia blood agar (CA). MBC was defined as the lowest concentration at which no bacterial growth was observed. The in vitro mode of antimicrobial action was interpreted based on the MBC/MIC ratio, using the following criteria: bactericidal if MBC/MIC ≤ 4 and bacteriostatic if MBC/MIC > 4 [28].

The effect of BTJ on aminoglycoside activity was assessed using the checkerboard broth microdilution method in accordance with established guidelines [29]. Fractional inhibitory concentration (FIC) values and the fractional inhibitory concentration index (FICI) were calculated to evaluate interactions [29]. Interpretation of FICI values was as follows: FICI ≤ 0.5, synergism; 0.5 < FICI ≤ 1, additive; 1 < FICI ≤ 4, indifference; FICI > 4, antagonism [30,31].

### 2.4. Statistical Analysis

The normality of data distribution within individual subgroups was assessed using the Shapiro–Wilk test. Since the same isolates from each species were employed to evaluate the effect of four antibiotics, a linear mixed model (LMM) was applied, with species and antibiotic as fixed factors and isolate as a random effect. Due to the right-skewed distribution, MIC values were log_2_-transformed. In post hoc analyses, planned pairwise comparisons of estimated marginal means were performed to assess differences in MIC between antibiotics within each bacterial species. *p*-alues were adjusted for multiple comparisons using the Bonferroni method to control for type I error. Statistical analyses were conducted in R (v. 4.3.2) using the lmerTest package (v. 3.1-3) for the omnibus test of MIC dependence on species and antibiotic, and emmeans (v. 1.10.5) for post hoc comparisons. The graph was generated using the ggplot2 package (v. 3.4.4).

## 3. Results

### 3.1. Characteristics of the Strains

The antimicrobial susceptibility profiles of the *Streptococcus* isolates, determined using the disc diffusion method in accordance with EUCAST 2025 guidelines, are presented in Table 2 [24].

Among the 30 clinical *Streptococcus* strains tested, five exhibited the MLS_B_ resistance mechanism (1 *S. pyogenes*, 4 *S. agalactiae*) while three showed the MS_B_ phenotype (2 *S. pyogenes*, 1 *S. agalactiae*). All macrolide-resistant strains were simultaneously resistant to tetracycline (T). In three additional strains, only resistance to T was observed. All other antibiotics tested demonstrated activity against the streptococcal isolates, with the exception of two strains: one *S. pneumoniae* isolate from blood and one *S. agalactiae* isolate from a cervical swab, both of which were resistant to norfloxacin. According to the adopted guidelines, the tested strains demonstrated susceptibility only to high concentrations of levofloxacin.

### 3.2. MICs of Aminoglycosides and β-Thujaplicin

**Figure 1:**
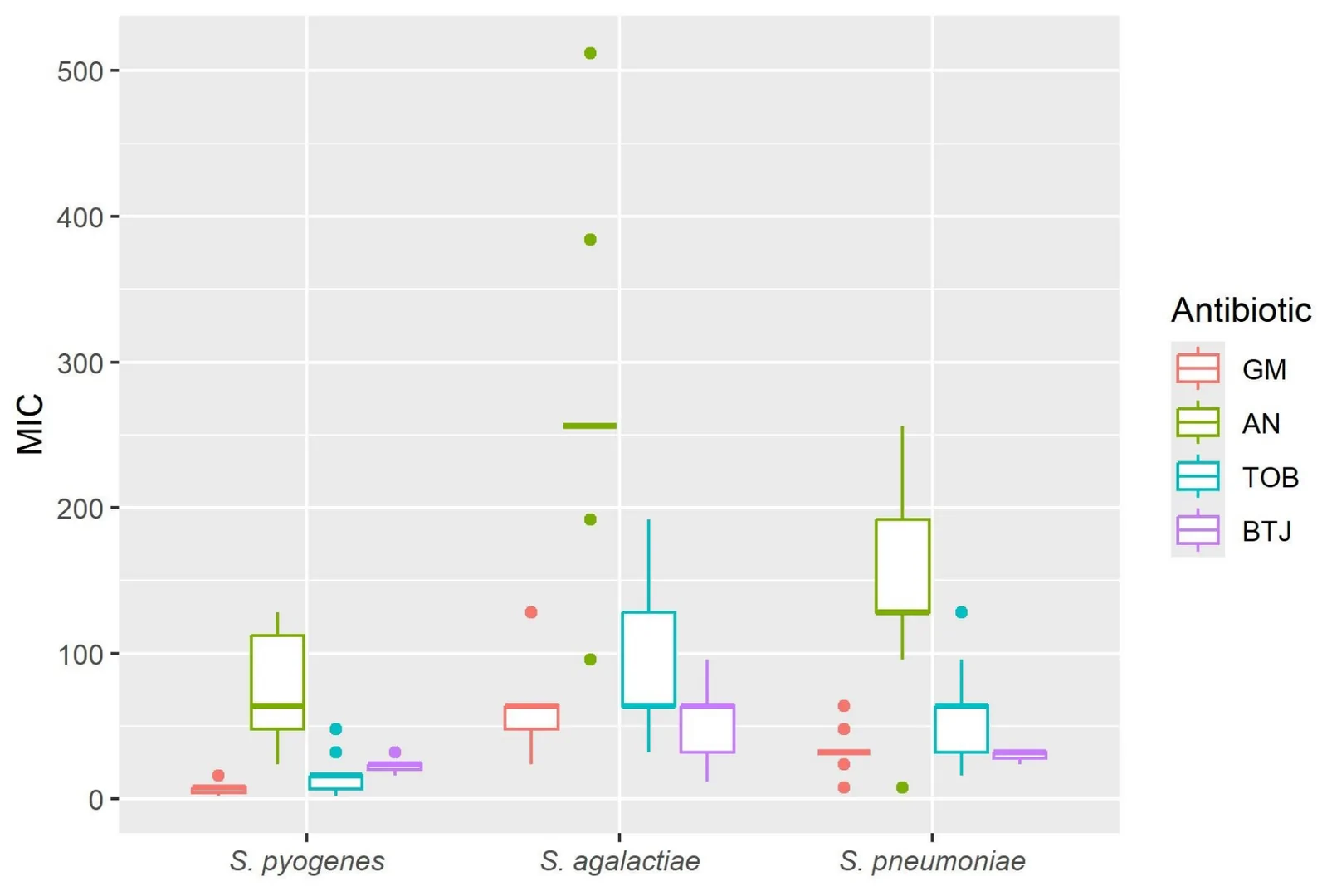
Comparison of the antimicrobial activity of gentamicin (GM), amikacin (AN), tobramycin (TOB) and β-thujaplicin (BTJ) against individual *Streptococcus* species, MIC—minimum inhibitory concentration [μg/mL]. The box-and-whisker plots present the median, the first and third quartiles (Q1 and Q3), the ranges (excluding outliers), and the outliers (values smaller than Q1—1.5 × IQR or larger than Q3 + 1.5 × IQR).

The MIC values of aminoglycosides and BTJ for both clinical and reference *Streptococcus* strains are provided in Table 3. The MIC value for DMSO was ≥12.5% (see Supplementary Materials Table S1). The antimicrobial susceptibility results for the additional reference strains—*Staphylococcus aureus* ATCC 29213 and *Enterococcus faecalis* ATCC 29212—as well as extended susceptibility data for *Streptococcus pneumoniae* ATCC 49619, are shown in Table 4. All MIC values for the reference strains fell within the recommended quality control ranges, confirming the accuracy and reliability of the testing procedure. Statistically significant effects of species, antibiotic, and the interaction species/antibiotic were observed (Table 5, Figure 1). The post hoc test results are presented in Table A1 (Appendix A).

Gentamicin and tobramycin exhibited the lowest MIC values against *Streptococcus* spp., with no significant difference between them in *S. agalactiae* (*p* = 0.4744). In the remaining species (*S. pyogenes*, *S. pneumoniae*), gentamicin was the most active of the tested aminoglycosides. Amikacin was significantly less effective than gentamicin (*p* < 0.0001) and tobramycin (*p* ≤ 0.0001) across all examined species (Figure 1).

The most sensitive species to GM was *S. pyogenes* (*S. pyogenes* vs *S. agalactiae*, *p* < 0.0001; *S. pyogenes* vs *S. pneumoniae*, *p* < 0.0001), with MIC_50_ and MIC_90_ values of 8 μg/mL and 16 μg/mL, respectively. According to EUCAST data on the distribution of gentamicin susceptibility in this species, the epidemiological cut-off value (ECOFF) is 32 μg/mL [27]. This indicates that the studied *S. pyogenes* strains can be classified as wild-type (WT ≤ 32 μg/mL) and phenotypically lacking detectable acquired resistance mechanisms to gentamicin. Higher MIC values were observed for TOB (MIC_50_ = 16 μg/mL, MIC_90_ = 32 μg/mL), and the highest for AN (MIC_50_ = 64 μg/mL, MIC_90_ = 128 μg/mL). The differences in antimicrobial activity among these antibiotics were statistically significant (*p* < 0.0001). As no ECOFFs have been established for TOB or AN in *S. pyogenes*, it is not possible to determine whether the elevated MICs reflect the intrinsic susceptibility of wild-type strains or the potential presence of acquired resistance mechanisms in non-wild-type isolates.

The weakest anti-streptococcal activity was found for AN, particularly against *S. agalactiae* (MIC_50_ = 256 μg/mL, MIC_90_ = 512 μg/mL) and *S. pneumoniae* (MIC_50_ = 128 μg/mL, MIC_90_ = 256 μg/mL). As in the case of *S. pyogenes*, it was not determined whether the tested isolates belonged to the wild type or non-wild-type according to EUCAST for this antibiotic.

Based on epidemiological cut-off values, two *S. agalactiae* isolates were identified as exhibiting a non-wild-type phenotype and may possess acquired resistance mechanisms to GM (MIC = 128 µg/mL). For TOB, no significant differences were observed (*p* = 0.2658) in the activity of this antibiotic against *S. agalactiae* and *S. pneumoniae* (MIC_50_ = 64 µg/mL and MIC_90_ = 128 µg/mL, respectively). However, two *S. agalactiae* isolates had TOB MIC values >128 µg/mL, classifying them as non-wild-type strains potentially harboring acquired resistance. One of these isolates displayed a non-wild-type phenotype with respect to both GM and TOB [27].

The range of BTJ concentrations inhibiting the growth of *S. pyogenes* and *S. pneumoniae* was narrower than that observed for aminoglycosides. For these species, BTJ inhibited growth at concentrations of 16 to 32 μg/mL, whereas for *S. agalactiae* strains the inhibitory range was broader, spanning 8 to 128 μg/mL. However, these differences were statistically significant only between *S. pyogenes* and *S. agalactiae* (*p* = 0.029). The highest MIC of BTJ (128 μg/mL) was found for the *S. agalactiae* non-wild-type strain against both GM and TOB.

BTJ activity against *S. pyogenes* was weaker than GM (*p* < 0.0001) and TOB (*p* = 0.0023), but stronger than AN (*p* < 0.0001). For *S. agalactiae* and *S. pneumoniae*, no significant differences were noted between the activity of BTJ and that of GM. BTJ also exhibited higher activity than AN across all *Streptococcus* species (*p* < 0.0001 for each comparison). The in vitro MBC/MIC ratios for BTJ (see Supplementary Materials, Table S2) suggest a potential bactericidal effect against all tested *S. pneumoniae* strains and against most strains of the other two streptococcal species. The exceptions were *S. pyogenes* 14 ST and 31 ST and *S. agalactiae* 10 ST, 15 ST, and 20 ST, for which it showed bacteriostatic activity.

### 3.3. Interaction Between β-Thujaplicin and Aminoglycosides

**Figure 2:**
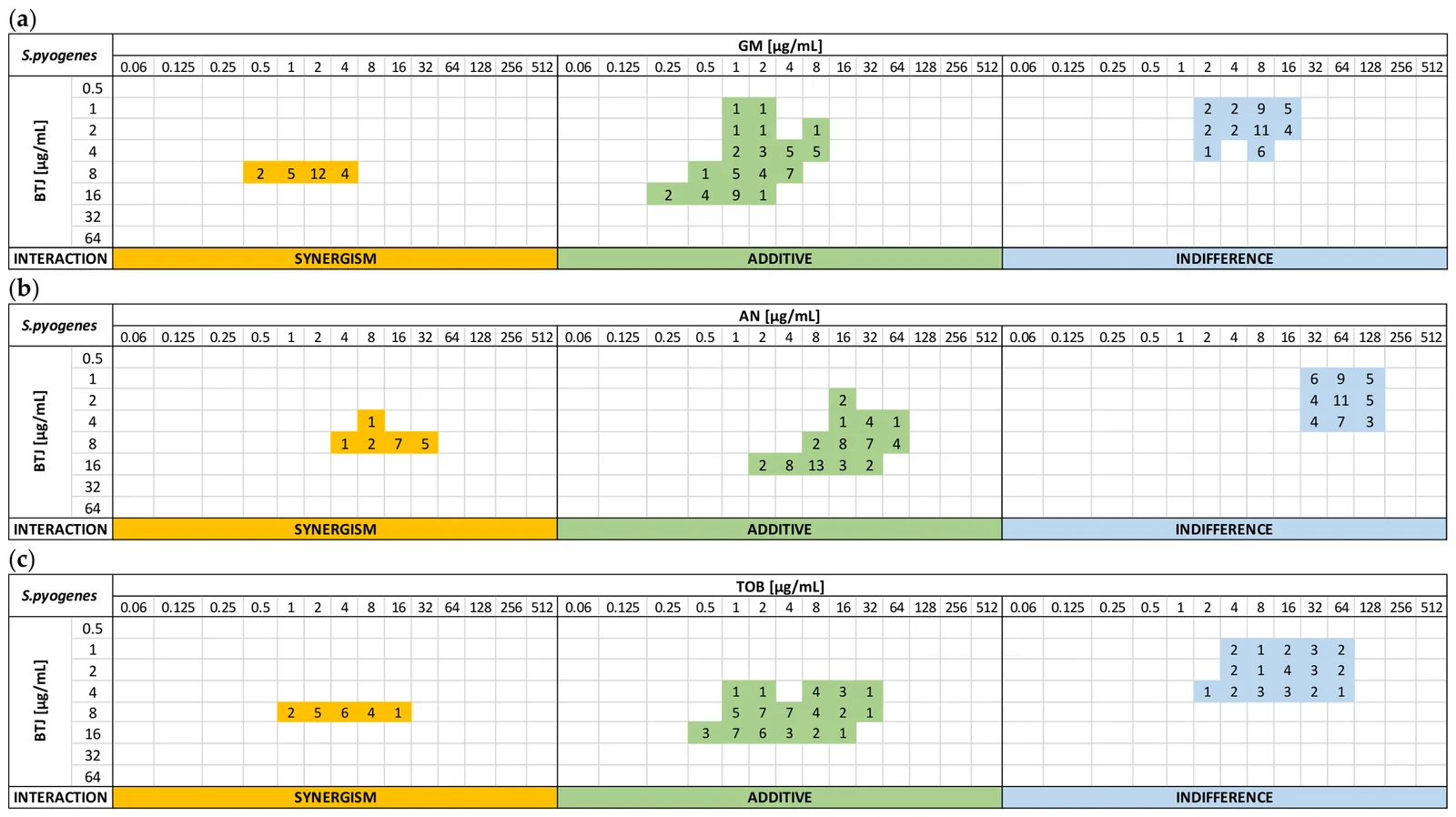
Number of synergistic, additive, or indifferent interactions between BTJ and (**a**) gentamicin (GM), (**b**) amikacin (AN), and (**c**) tobramycin (TOB) in *S. pyogenes* strains.

**Figure 3:**
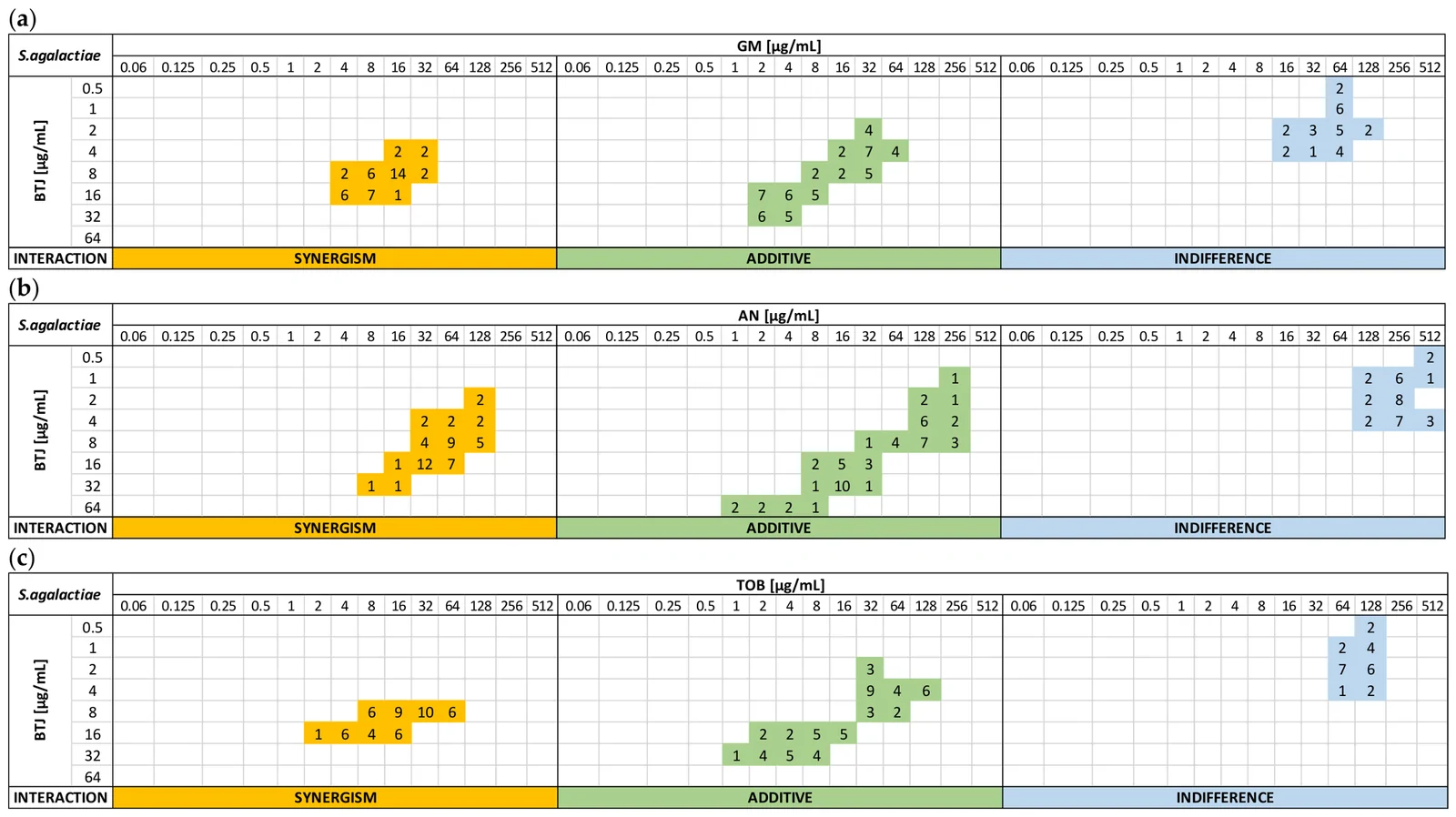
Number of synergistic, additive, or indifferent interactions between BTJ and (**a**) gentamicin (GM), (**b**) amikacin (AN), and (**c**) tobramycin (TOB) in *S. agalactiae* strains.

**Figure 4:**
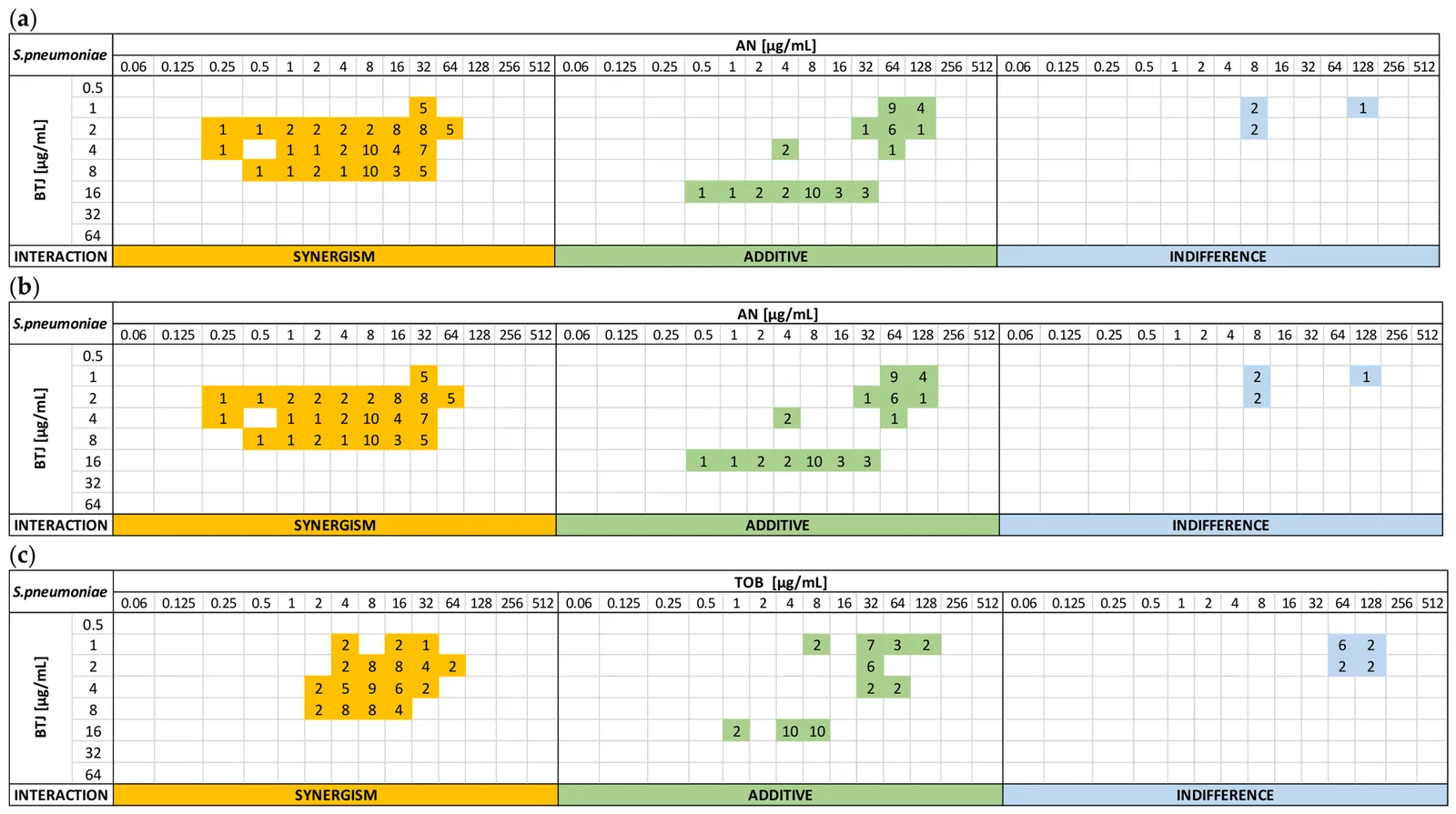
Number of synergistic, additive, or indifferent interactions between BTJ and (**a**) gentamicin (GM), (**b**) amikacin (AN), and (**c**) tobramycin (TOB) in *S. pneumoniae* strains.

The type of interaction between the various concentrations of BTJ and the concentrations of the individual antibiotics is illustrated by Figure 2, Figure 3 and Figure 4, which present results from two independent measurements.

For *S. pyogenes* isolates, synergistic and additive interactions between BTJ and aminoglycosides were noted at a BTJ concentration of 8 μg/mL, which is 2- to 4-fold lower than the BTJ MIC. The synergistic effect of BTJ contributed to a reduction in MIC values for GM from 2–16 μg/mL to 0.5–4 μg/mL, for AN from 16–128 μg/mL to 4–32 μg/mL, and for TOB from 2–64 μg/mL to 1–16 μg/mL (Table 3, Figure 2a–c). Since acquired resistance mechanisms to GM were not suspected in *S. pyogenes*, these findings suggest that BTJ can enhance aminoglycoside susceptibility in isolates lacking phenotypically detectable non-wild-type characteristics. No antagonistic interactions were detected within the tested concentration range. As the BTJ concentration decreased below 8 μg/mL, an increased frequency of indifferent interactions with the antibiotics was noted. The concentration ranges at which synergistic interactions between the two agents were observed against *S. pyogenes*, along with the corresponding proportions of each compound, are presented in Table 6.

Synergism was typically associated with BTJ concentrations 2–16 times higher than those of GM, and equal to or 2–8 times higher than TOB. In contrast, when combined with AN, the antibiotic concentration was most often 2–4 times higher than that of BTJ. Synergistic interactions between BTJ and each of the aminoglycosides were found in 50% of the clinical *S. pyogenes* isolates tested. In 20% of isolates, no synergy was detected in any combination; however, additive effects were observed for selected BTJ–antibiotic concentration pairs in all isolates. These findings did not appear to be associated with other antibiotic resistance mechanisms, such as MLS_B_, MS_B_, or tetracycline resistance. No isolates exhibited exclusively indifferent interactions.

In *S. agalactiae* isolates, synergy between BTJ and aminoglycosides occurred across a broader range of BTJ concentrations than in *S. pyogenes* (Figure 3).

The highest number of synergistic interactions was detected at BTJ concentrations of 8–16 μg/mL, which are below the MIC value for 90% of the tested isolates. The synergistic activity of BTJ contributed to a reduction in MIC values for GM from 16–128 μg/mL to 4–32 μg/mL, for AN from 64–512 μg/mL to 8–128 μg/mL, and for TOB from 32–256 μg/mL to 2–64 μg/mL (Table 3, Figure 3a–c). Additive effects were also noted across a broader range of aminoglycoside and BTJ concentrations, similar to the pattern seen in *S. pyogenes.* Indifference was detected only at BTJ MIC values ≤ 4 μg/mL. No antagonistic interactions were observed.

BTJ also reduced MIC (GM) in two isolates classified as non-wild-type according to ECOFFs. In the presence of BTJ at 8 and 16 μg/mL, MIC (GM) decreased from 128 μg/mL to 4–32 μg/mL, resulting in a change in classification to the wild-type category. In one of these isolates, the same BTJ concentrations also reduced MIC (TOB) from 256 μg/mL to 8–64 μg/mL, a value below the corresponding ECOFF.

The BTJ and aminoglycoside concentration ranges that produced synergistic interactions against *S. agalactiae*, along with the corresponding proportions of each compound, are provided in Table 7.

Synergy between BTJ and GM was found in 80% of clinical isolates, with AN in 100% and with TOB in 90% of isolates. As in *S. pyogenes*, the interaction between BTJ and AN was associated with the use of antibiotic concentrations 2–16 times higher than those of BTJ. The proportions in BTJ–GM and BTJ–TOB combinations were more variable. In individual isolates, synergistic activity often occurred both when the antibiotic concentration exceeded that of BTJ and when the opposite was true. No evidence was found that these patterns were associated with coexisting acquired resistance mechanisms to macrolides, lincosamides, or tetracyclines, nor with the MIC values of the antibiotics or BTJ.

Synergy between BTJ and a broad range of aminoglycoside concentrations was observed in *S. pneumoniae* isolates (Figure 4).

In the presence of aminoglycosides, the MIC (BTJ) decreased from 16 to 32 μg/mL to 1–8 μg/mL. Conversely, BTJ activity contributed to a reduction in MIC(GM) from 8 to 64 μg/mL to 0.06–16 μg/mL, in MIC(AN) from 8 to 256 μg/mL to 0.025–64 μg/mL, and in MIC(TOB) from 16 to 128 μg/mL to 2–64 μg/mL (Table 3, Figure 4). Indifference was observed only at BTJ MIC values ≤ 2 μg/mL. No antagonistic interactions were detected. The BTJ and aminoglycoside concentration ranges that resulted in synergistic activity against *S. pneumoniae*, along with the corresponding proportions of each compound, are presented in Table 8.

In contrast to the other streptococcal species examined, synergistic activity was noted in all *S. pneumoniae* isolates, regardless of the compound combination used. Synergy occurred most frequently at BTJ concentrations of 4–8 μg/mL, which is 4-fold lower than the MIC of this compound for *S. pneumoniae*. In combination with AN and TOB, synergy was generally associated—aside from isolated exceptions—with the use of antibiotic concentrations equal to or higher than those of BTJ. In combinations with gentamicin, these proportions were more variable, even within individual isolates.

## 4. Discussion

Currently, streptococcal species with high pathogenic potential and clinical relevance in human infections (*S. pyogenes*, *S. agalactiae*, *S. pneumoniae*) have been classified as a medium group on the WHO list of priority bacterial threats. They have been recognized as microorganisms for which new therapeutic options should be sought, in part due to their increasing antimicrobial resistance [14]. For this reason, the present study aimed to assess the effect of β-thujaplicin (BTJ), a compound with potential antibacterial activity, on this group of bacteria and to determine whether it exhibits synergistic interactions with aminoglycosides, to which these species naturally have reduced susceptibility.

The activity of BTJ against *Streptococcus* spp. has been only sporadically reported since the 1970s by a limited number of researchers [2,5,12,32,33,34,35,36]. The diverse methodologies used in those studies and the heterogeneous results—typically presented for single isolates from different *Streptococcus* species—did not allow for a clear assessment of BTJ activity or meaningful comparison with antibiotic effects. The present work provides results for the largest collection of isolates studied to date, both reference strains and clinical isolates derived from infections, including invasive disease. MIC values for BTJ, aminoglycosides, and their combined activity based on the FICI were determined, the latter being reported here for the first time in the scientific literature.

Among the clinical streptococcal isolates examined here, a significant proportion exhibited multidrug resistance (MDR). This was most evident in *S. agalactiae*, of which 50% were resistant to macrolides, 40% additionally displayed resistance to lincosamides and streptogramins (MLS_B_ phenotype), and 90% were simultaneously resistant to tetracycline. A lower proportion of MDR isolates was noted for *S. pyogenes* (10%), where similar MLS_B_ and tetracycline resistance mechanisms were detected. In contrast, *S. pneumoniae* isolates showed resistance only to norfloxacin, identified in a single bloodstream isolate.

When assessing the susceptibility of *Streptococcus* spp. to aminoglycosides, isolates of *S. agalactiae* were identified that exhibited an elevated level of intrinsic resistance to this class of antibiotics. Comparison of MIC values with the ECOFF for gentamicin revealed two isolates of this species that could not be classified as wild type. Hays et al., applying the Antimicrobial Susceptibility Testing guidelines of the French Society for Microbiology, observed an increase in the prevalence of *S. agalactiae* strains with high-level gentamicin resistance (HLGR)—highly resistant to AN and GM (MIC > 128 µg/mL)—from 6.4% to 8.8% between 2007 and 2014. This phenotype resulted from the presence of a genomic sequence encoding a bifunctional enzyme with 6′-acetyltransferase and 2″-phosphotransferase activity [37]. Similarly, Doumith et al. identified *S. agalactiae* isolates with MIC(GM) > 1024 µg/mL, in which the *aac(6′)-aph(2″)* encoding these enzymes were located on transposons [38]. In the present study, *S. agalactiae* isolates with such high MIC(GM) values were not detected (MIC_50_ = 64 μg/mL, MIC_90_ = 128 μg/mL), and genomic analysis of these strains will be the subject of future investigations.

In contrast, the results for *S. pyogenes* are difficult to compare with those of other researchers, as aminoglycoside susceptibility testing with MIC determination has not been routinely performed for this species for many years. Studies from the past 25 years typically report only qualitative assessments using the disc diffusion method [39]. In 2021, Moreno et al., using an E-test strip, determined the MIC (GM) for the reference strain *S. pyogenes* ATCC 19615 to be 1 μg/mL, which was slightly lower than that in the present study (Table 3). This difference may, however, reflect variations in the applied methodological approaches [20].

Similarly, *S. pneumoniae* isolates in this study were less susceptible to GM (MIC_50_ = 32 μg/mL, MIC_90_ = 64 μg/mL) compared with the findings of Wohlfarth et al., who reported that isolates obtained from ocular and conjunctival swabs between 2004 and 2024 did not exceed a GM MIC of 8 μg/mL [40]. Marks et al. reported MIC values of 16 μg/mL for four isolates of this species [41].

Evaluation of the anti-streptococcal activity of BTJ suggests that this compound may exhibit bactericidal properties under the tested in vitro conditions, as indicated by the MBC/MIC ratio. For individual strains, however, this effect was only bacteriostatic. BTJ inhibited the growth of *S. agalactiae* at concentrations of 8–128 μg/mL and the growth of *S. pyogenes* and *S. pneumoniae* at concentrations of 16–32 μg/mL. High bactericidal activity of BTJ had previously been suggested by Domon et al., who demonstrated that incubation of *S. pneumoniae* at a concentration corresponding to the MIC (BTJ) resulted in a >99% reduction in bacterial viability within an hour [5]. The activity of BTJ in the present study was stronger than that of amikacin across all tested species and comparable to gentamicin in *S. agalactiae* and *S. pneumoniae*, which may indicate that BTJ acts within a similar concentration range to gentamicin, although its mechanism of action may differ. An exception was noted for *S. pyogenes*, whose growth was more strongly inhibited by gentamicin than by BTJ. At the same time, MIC(BTJ) values for both clinical and reference strains were similar, suggesting that the phenotypically detected acquired resistance mechanisms in the tested streptococci do not significantly influence the activity of BTJ.

The available literature on the effects of BTJ on *Streptococcus spp*. is limited. In 1973, Trust et al. were among the first to evaluate the antimicrobial activity of BTJ against Gram-positive bacteria, including streptococci. Using a well-diffusion assay and three selected concentrations of the compound, they demonstrated that S*. pyogenes* was resistant to the lowest concentration tested (31 μg/mL), whereas *S. pneumoniae* showed greater susceptibility [32]. Subsequently, Saeki et al. confirmed the antibacterial activity of BTJ against oral streptococci [33]. Domon et al. later determined MIC values for BTJ using the microdilution method, reporting an MIC of 0.3 μg/mL for *S. pyogenes* and a range of 0.3–1 μg/mL for four *S. pneumoniae* isolates, suggesting substantially greater susceptibility than reported in the present study [5]. However, the microdilution method used by those authors differed from the EUCAST-based methodology applied here for antibiotic susceptibility testing. In Domon et al., bacteria were incubated under aerobic conditions in casein–soy broth (*S. pneumoniae*), or Todd–Hewitt broth (*S. pyogenes*), and the absence of a microaerophilic atmosphere—favorable for streptococcal growth—may have rendered the bacteria more susceptible to the tested compound [5].

In 2021, Houdková et al., using a custom macrodilution method, assessed the therapeutic potential of BTJ in both liquid and vapor phases, determining MIC values of 64 μg/mL for S*. pyogenes* and *S. pneumoniae* in liquid form and 1024 μg/mL in vapor form [34]. These values were higher than those reported in the present study, likely due to differences in the preparation and dilution of BTJ solutions. The activity of BTJ against other streptococci was evaluated by Wang et al., but only for a single *S. mutans* isolate, for which the MIC (BTJ) was 40 μg/mL [35]. Shih et al. found MIC (BTJ) values of 70–80 μg/mL for this species using various dilution methods [2]. Sensitivity of *Streptococcus bovis* at 1.56 μg/mL was described by Ishii et al. [36].

The only in vivo study investigating the effect of BTJ on streptococcal infection was conducted by Isono et al. using a murine antimicrobial-resistant pneumococcal pneumonia model. The authors confirmed the efficacy of BTJ in reducing *S. pneumoniae* counts in bronchoalveolar lavage fluid and decreasing bacterial DNA levels in serum, indicating that this compound may represent a potential alternative to antibiotics [12]. Notably, BTJ demonstrated comparable efficacy against both macrolide-susceptible and macrolide-resistant strains, supporting the notion that its mechanism of action differs from that of macrolides.

One of the proposed mechanisms of BTJ activity in bacterial cells involves damage to the cell wall and cytoplasmic membrane [8]. Differences in cell envelope structure may underlie the varying susceptibility of Gram-positive and Gram-negative bacteria to this compound [22]. BTJ also acts as a chelator of divalent metal ions such as zinc (Zn), copper (Cu), and iron (Fe), which are essential for the activity of metallo-dependent enzymes. In addition, it may influence protein synthesis and electron transport in the respiratory chain, although its precise mechanism of action in bacterial cells has not yet been fully elucidated [7,8].

Owing to its multifaceted activity, BTJ may enhance the efficacy of antibiotics and slow the development of antimicrobial resistance. Le et al. were the first to demonstrate this potential in vitro, showing that BTJ acts synergistically with tetracycline against both susceptible and resistant *Staphylococcus aureus* strains [42]. At a concentration of 1 μg/mL, BTJ reduced the MIC of tetracycline in staphylococci by 4- to 64-fold by increasing intracellular accumulation of the antibiotic. Using EUCAST criteria, it can be concluded that in one of the strains BTJ additionally restored in vitro susceptibility to tetracycline [24]. Synergistic interactions between BTJ and tetracycline derivatives have also been observed in the Gram-negative bacterium *Escherichia coli* [43]. When used in vitro as an adjuvant in combination with tigecycline, BTJ exhibited synergistic activity against a strain carrying the *tet*(X4) resistance gene.

The administration of combination therapy involving two or more antimicrobial agents has become an essential strategy in the era of increasing antimicrobial resistance [44,45,46]. An alternative therapeutic approach may involve combining antibiotics with novel compounds or nanoparticles that interfere with various metabolic processes in bacterial cells and act as inhibitors of both acquired and intrinsic resistance mechanisms. BTJ has such properties, although to date they have been demonstrated only in methicillin-resistant *Staphylococcus aureus* strains when used in combination with bismuth-based compounds [47]. In *Streptococcus*, several compounds have been found to act synergistically with aminoglycosides, and the results presented here indicate for the first time that BTJ may be one of them [48,49]. While the antibacterial activity of BTJ against various bacterial species has been relatively well documented in the literature, reports on its use in combination with antibiotics to enhance bacterial susceptibility remain scarce [2,5,8].

In the present study, gentamicin was identified as the most active aminoglycoside against *Streptococcus* spp., with *S. pyogenes* being the most susceptible species. BTJ exhibited activity comparable to gentamicin in *S. agalactiae* and *S. pneumoniae* and demonstrated significantly greater efficacy than amikacin across all streptococcal strains. The only exception was the higher activity of gentamicin compared with BTJ against *S. pyogenes*. Among *S. agalactiae* isolates, two strains were identified with elevated aminoglycoside MIC values, potentially indicating the presence of acquired resistance mechanisms. However, confirmation of this hypothesis requires further molecular analysis.

This study demonstrated a synergistic interaction between BTJ and aminoglycosides, despite the fact that these antibiotics are generally not considered therapeutic options for infections caused by *Streptococcus pneumoniae*, *S. pyogenes*, or *S. agalactiae* due to their intrinsic resistance to low concentrations of these agents. In the presence of BTJ, most commonly at a concentration of 8 μg/mL, a 2- to 64-fold increase in susceptibility to aminoglycosides was observed. Additionally, in *S. agalactiae* strains with high MIC values for GM and TOB, classified according to ECOFFs as non-wild-type, BTJ reduced the MICs of these antibiotics and changed their classification to a wild-type phenotype. A plausible mechanism underlying this effect may involve BTJ-induced damage to the cell wall or cytoplasmic membrane, leading to increased intracellular access of high-molecular-weight aminoglycosides, similar to the enhancement observed in the presence of β-lactams or rifampicin [20]. However, this hypothesis requires further investigation.

Synergy varied among streptococcal species. In *S. pneumoniae*, synergistic effects were observed across the broadest range of concentration combinations and were present in 100% of the isolates tested, compared with 50% of *S. pyogenes* and 80% of *S. agalactiae* clinical isolates. It is noteworthy that additive effects were detected in all strains examined. The finding that not all streptococcal isolates respond equally to the same concentration combinations suggests the presence of additional, yet unidentified strain-specific factors influencing BTJ activity.

Future studies should also clarify the dependence of synergy on the relative proportions of BTJ and individual aminoglycosides. Synergy between BTJ and amikacin typically occurred at antibiotic concentrations 2- to 64-fold higher than those of BTJ, in contrast to BTJ–GM or BTJ–TOB combinations. Species-specific differences were also evident. Importantly, the combination of BTJ with aminoglycosides reduced not only the MIC of the antibiotics but also that of BTJ itself.

The ability to lower the effective concentrations of both synergistic agents is a key advantage of combination therapy, as it reduces the likelihood of adverse toxic effects and decreases the risk of resistance development —an especially important consideration in the treatment of multidrug-resistant pathogens [45]. The significance of BTJ as a compound with such properties requires in vivo confirmation, which was beyond the scope of the present study.

The potential use of BTJ as an adjuvant in combination with aminoglycosides requires extensive further research, including evaluation of anti-biofilm activity, physicochemical compatibility between compounds, pharmacokinetic and pharmacodynamic interactions, and the impact of BTJ on the post-antibiotic effect of aminoglycosides. Equally important is the assessment of cytotoxicity of such combinations. However, BTJ itself is of low toxicity, stable across a wide range of temperatures and pH values, and capable of retaining its antibacterial properties even after photolysis [5,10,35,50]. These properties have already led to its incorporation into products for the prevention and treatment of oral infections such as toothpastes, mouth rinses, and care gels used in the EU, the United States, and Japan [2,5,51,52,53]. Nevertheless, the potential application of BTJ in combination with gentamicin, for example in *Streptococcus*-associated infective endocarditis, should not be excluded. A recent study by Karunakar et al. demonstrated that BTJ may also confer protection against gentamicin-induced renal injury by attenuating oxidative stress and modulating inflammatory pathways [54]. Such an application, however, will require further investigation, of which the present study represents an initial step.

## 5. Limitations

This study has several limitations that should be taken into account when interpreting its findings. First, only in vitro experiments have been performed to date, and their results cannot be directly extrapolated to potential in vivo effects. The preliminary assessment of the bactericidal activity of BTJ should be verified using additional methods, such as time-kill assays, because the MBC/MIC ratio is an indirect measure of this property. It also remains unclear why BTJ exhibits bacteriostatic activity in individual strains, which warrants further investigation. Moreover, evaluating the activity of BTJ against biofilm forms of microorganisms—an important contributor to human infections—would be valuable and will be addressed in future studies.

Another limitation in interpreting the results is the inability to classify some isolates as wild-type or non-wild-type, as EUCAST has not established ECOFFs for certain antibiotics. Furthermore, this study employed only two non-wild-type isolates. However, the next stage of the research will seek to determine the reasons for their elevated MIC values for selected aminoglycosides and investigate the mechanisms underlying their synergistic interactions with aminoglycosides.

## 6. Conclusions

Using the largest collection of clinical isolates examined to date, this study demonstrated that BTJ exhibits antimicrobial activity against *S. pyogenes* and *S. pneumoniae* (MIC 16–32 µg/mL) as well as *S. agalactiae* (MIC 8–128 µg/mL). In vitro, BTJ was more effective than amikacin and showed indications of bactericidal activity against most isolates. BTJ also displayed synergistic interactions with aminoglycosides in a substantial proportion of strains (*S. pneumoniae*—100%, *S. agalactiae*—80%, *S. pyogenes*—50%), leading to a 2–64-fold reduction in aminoglycoside MIC values, most commonly at a BTJ concentration of 8 µg/mL. However, explanation of the mechanism underlying this synergistic interaction and determination of the optimal BTJ/antibiotic ratios will require further studies, both in vitro and subsequently in vivo.

## Figures and Tables

**Table 1:** Clinical origin of *Streptococcus* spp. strains.

Species	Type of Material							
	Wound Swab	Pus	Cervical Swab	Ear Swab	Throat/Tonsils Swab	Bronchial Secretion	Blood	Urine
*S. pyogenes*	4	3	-	-	2	1	-	-
*S. agalactiae*	5	-	3	-	1	-	-	1
*S. pneumoniae*	-	-	-	1	1	1	7	-

**Table 2:** Number of *Streptococcus* strains categorized as susceptible (S), resistant (R), or susceptible with increased exposure (I) according to EUCAST guidelines [24].

Species	P/OX ^a^	E	CC	T	TGC	SXT	NOR	MXF	LVX	VA	TEC	LZD	RIF
*S. pyogenes* (*n* = 10)	S	S(7) R(3)	S(9) R(1)	S(6) R(4)	S	S	S	S	I	S	S	S	S
*S. agalactiae* (*n* = 10)	S	S(5) R(5)	S(7) R(3)	S(1) R(9)	S	S	S(9) R(1)	S	I	S	S	S	S
*S. pneumoniae* (*n* = 10)	S	S	S	S	S	S	S(9) R(1)	S	I	S	S	S	S

^a^ penicillin (P) for *S. pyogenes* and *S. agalactiae*, oxacillin (OX) screening for *S. pneumoniae*, erythromycin (E), clindamycin (CC), tetracycline (T), tigecycline (TGC), trimethoprim/sulfamethoxazole (SXT), norfloxacin (NOR), moxifloxacin (MXF), levofloxacin (LVX), vancomycin (VA), teicoplanin (TEC), linezolid (LZD), rifampicin (RIF).

**Table 3:** MIC values of gentamicin (GM), amikacin (AN), tobramycin (TOB) and β-thujaplicin (BTJ) for the tested strains of *Streptococcus* spp. Abbreviations: I—first measurement, II—second measurement, $\bar{x}$—mean MIC value.

Species	Strain Number	MIC [μg/mL]											
		GM			AN			TOB			BTJ		
		I	II	$\bar{x}$	I	II	$\bar{x}$	I	II	$\bar{x}$	I	II	$\bar{x}$
*Streptococcus pyogenes*	9ST	16	16	16	128	128	128	64	32	48	32	16	24
	14ST	8	8	8	64	64	64	8	8	8	32	16	24
	18ST	8	8	8	64	64	64	16	16	16	16	16	16
	26ST	2	2	2	16	32	24	2	2	2	16	16	16
	27ST	4	2	3	32	32	32	8	4	6	32	16	24
	28ST	8	8	8	128	128	128	16	16	16	32	16	24
	29ST	8	8	8	64	128	96	16	16	16	32	32	32
	30ST	16	16	16	128	128	128	32	32	32	32	16	24
	31ST	2	2	2	32	64	48	2	4	3	32	16	24
	50ST	8	8	8	64	64	64	16	16	16	16	16	16
	ATCC 19615	4	8	6	32	64	48	8	16	12	32	16	24
	MIC Range	2–16			16–128			2–64			16–32		
*Streptococcus agalactiae*	10ST	128	128	128	512	512	512	128	256	192	128	64	96
	11ST	64	64	64	256	256	256	256	128	192	64	64	64
	12ST	64	64	64	256	512	384	128	64	96	16	8	12
	13ST	64	64	64	256	256	256	128	128	128	64	64	64
	15ST	32	16	24	128	64	96	32	32	32	32	32	32
	19ST	64	64	64	256	256	256	64	64	64	32	32	32
	20ST	32	32	32	128	256	192	32	32	32	64	64	64
	51ST	128	128	128	256	256	256	64	64	64	64	64	64
	55ST	64	64	64	256	256	256	128	128	128	32	32	32
	56ST	64	64	64	256	256	256	64	64	64	64	64	64
	ATCC 12386	32	32	32	256	256	256	64	64	64	32	32	32
	MIC Range	16–128			64–512			32–256			8–128		
*Streptococcus pneumoniae*	17ST	32	32	32	128	128	128	64	64	64	32	16	24
	21ST	32	32	32	128	256	192	64	64	64	32	32	32
	22ST	64	64	64	256	256	256	64	128	96	32	32	32
	23ST	32	32	32	64	128	96	32	32	32	16	32	24
	24ST	32	64	48	128	256	192	32	32	32	32	32	32
	25ST	16	32	24	128	256	192	32	32	32	32	16	24
	35ST	32	32	32	256	256	256	128	128	128	32	32	32
	42ST	32	32	32	128	128	128	64	64	64	32	32	32
	46ST	32	32	32	128	128	128	64	64	64	32	32	32
	47ST	32	32	32	128	128	128	64	64	64	32	32	32
	ATCC 49619	8	8	8	8	8	8	16	16	16	32	32	32
	MIC Range	8–64			8–256			16–128			16–32		

**Table 4:** MIC values of gentamicin (GM), amikacin (AN), tobramycin (TOB), erythromycin (E), and tetracycline (T) for the reference strains tested.

Strain	MIC [μg/mL]				
	GM [Range]	AN [Range]	TOB [Range]	E [Range]	T [Range]
*S. aureus* ATCC 29213	0.5 [0.125–1]	2 [1,2,3,4]	0.125 [0.125–1]	0.5 [0.25–1]	0.25 [0.125–1]
*E. faecalis* ATCC 29212	8 [4,5,6,7,8,9,10,11,12,13,14,15,16]	- *	-	-	-
*S. pneumoniae* ATCC 49619	-	-	-	0.03 [0.03–0.125]	0.06 [0.06–0.5]

* The susceptibility of reference strains to antibiotics for which EUCAST does not provide quality control ranges or targets was not determined.

**Table 5:** The effect of species and antibiotic on the aminoglycoside and the BTJ activity in a linear mixed model. Abbreviations: df—degrees of freedom, F—test statistics, *p*—statistical significance.

Effect	df	F	*p*
Antibiotic	3, 90	103.15	<0.0001
Species	2, 30	29.14	<0.0001
Antibiotic × Species	6, 90	9.02	<0.0001

**Table 6:** Summary of the synergistic activity of β-thujaplicin (BTJ) in combination with aminoglycosides (GM, AN, TOB) against *S. pyogenes*.

*S. pyogenes*	GM [μg/mL]	BTJ [μg/mL]	Proportion GM/BTJ	AN [μg/mL]	BTJ [μg/mL]	Proportion AN/BTJ	TOB [μg/mL]	BTJ [μg/mL]	Proportion TOB/BTJ
9ST	2–4	8	1:2 1:4	32	8	4:1	-	-	-
14ST	0.5–2	8	1:4 1:8 1:16	16	8	2:1	1–2	8	1:2 1:4 1:8
18ST	-	-	-	-	-	-	-	-	-
26ST	-	-	-	4–8	4–8	1:1 1:2 2:1	-	-	-
27ST	1	8	1:8	-	-	-	1–2	8	1:4 1:8
28ST	2–4	8	1:2 1:4	8–32	8	1:1 2:1 4:1	2–8	8	1:1 1:2 1:4
29ST	2	8	1:4	16	8	2:1	4–8	8	1:1 1:2
30ST	2–4	8	1:2 1:4	32	8	4:1	4–16	8	1:1 1:2
31ST	0.5–1	8	1:8 1:16	-	-	-	-	-	-
50ST	-	-	-	-	-	-	-	-	-
ATCC 19615	1–2	8	1:4 1:8	16	8	2:1	2–4	8	1:2 1:4

**Table 7:** Summary of the synergistic activity of β-thujaplicin (BTJ) in combination with aminoglycosides (GM, AN, TOB) against *S. agalactiae*.

*S. agalactiae*	GM [μg/mL]	BTJ [μg/mL]	Proportion GM/BTJ	AN [μg/mL]	BTJ [μg/mL]	Proportion AN/BTJ	TOB [μg/mL]	BTJ [μg/mL]	Proportion TOB/BTJ
10ST	4–32	8–16	1:1 1:2 4:1	8–128	8–32	1:2 4:1 16:1	8–64	8–16	1:1 1:2 8:1
11ST	8–16	8–16	1:2 2:1	32–128	8–16	2:1 4:1 16:1	16–64	8–16	1:1 4:1 8:1
12ST	-	-	-	128	2–4	32:1 64:1	-	-	-
13ST	4–16	8–16	1:1 2:1 1:4	32–128	8–16	4:1 8:1 16:1	16–64	8–16	1:1 4:1 8:1
15ST	-	-	-	32	16	2:1	16	8	2:1
19ST	8–16	8	1:1 2:1	32–128	8–16	2:1 8:1 16:1	32	8	4:1
20ST	16	8	2:1	64	8	8:1	4–16	8–16	1:2 1:4 2:1
51ST	8–32	4–16	1:2 2:1 8:1	32–64	8–16	2:1 8:1	2–16	8–16	1:1 1:8 2:1
55ST	16	4–8	2:1 4:1	32–64	4–8	4:1 8:1 16:1	32	8	4:1
56ST	4–16	8–16	1:2 1:4	32–64	8–16	2:1 8:1	4–16	8–16	1:1 1:4
ATCC 12386	4–8	8	1:1 1:2	32–64	8	4:1 8:1	8–16	8	1:1 2:1

**Table 8:** Summary of the synergistic activity of β-thujaplicin (BTJ) in combination with aminoglycosides (GM, AN, TOB) against *S. pneumoniae*.

*S. pneumoniae*	GM [μg/mL]	BTJ [μg/mL]	Proportion GM/BTJ	AN [μg/mL]	BTJ [μg/mL]	Proportion AN/BTJ	TOB [μg/mL]	BTJ [μg/mL]	Proportion TOB/BTJ
17ST	0.5–8	4–8	1:1 1:8 1:16	2–32	1–8	1:1 8:1 16:1	4–32	2–8	1:1 2:1 16:1
21ST	4–16	2–8	1:2 4:1 8:1	32–64	2–8	4:1 8:1 32:1	8–32	1–8	1:1 8:1 32:1
22ST	4–16	4–8	1:1 1:2 4:1	32–64	2–8	4:1 8:1 32:1	16–32	4–8	2:1 8:1
23ST	1–8	2–8	1:1 1:4 2:1	8–32	2–8	1:1 4:1 16:1	4–16	2–8	1:1 4:1 8:1
24ST	4–8	4–8	1:1 1:2 2:1	16–32	4–8	2:1 8:1	8–16	4–8	1:1 4:1
25ST	0.25–8	2–8	1:1 1:8 1:16	0.25–32	1–8	1:4 1:8 32:1	4–16	2–8	1:1 1:2 8:1
35ST	2–8	4–8	1:2 1:4 2:1	8–64	2–8	1:1 2:1 32:1	8–64	2–8	1:1 16:1 32:1
42ST	4–8	4–8	1:2 2:1	16–32	4–8	2:1 8:1	16	8	2:1
46ST	0.06–8	2–8	1:1 1:16 1:32	8–32	1–8	1:1 16:1 32:1	8–16	2–8	1:1 2:1 8:1
47ST	2–8	2–8	1:4 2:1 4:1	4–32	1–8	1:2 8:1 32:1	4–16	1–8	2:1 4:1 16:1
ATCC 49619	2	8	1:4	2	8	1:4	2–4	1–8	1:2 1:4 4:1

## Data Availability

The datasets generated during and/or analyzed during the current study are not publicly available due to the fact that the research data constitute an integral component of an ongoing project, and their premature public release could disrupt the proper conduct of the research process, but are available from the corresponding author upon reasonable request.

## Supplementary Materials

The following supporting information can be downloaded at: https://www.mdpi.com/article/10.3390/pathogens15090978/s1. Table S1: MIC values of DMSO (BTJ solvent); Table S2: MBC/MIC ratios for antibiotics and BTJ in the tested strains: (a) *S. pyogenes*, (b) *S. agalactiae*, (c) *S. pneumoniae*; I—first measurement, II—second measurement.

## Abbreviations

The following abbreviations are used in this manuscript:

AN	Amikacin
ATCC	American Type Culture Collection
BHI	Brain–heart infusion broth
β-NAD	β-nicotinamide adenine dinucleotide
BTJ	β-thujaplicin
CA	Columbia agar
CC	Clindamycin
Cu	Copper
df	Degrees of freedom
DMSO	Dimethyl sulphoxide
E	Erythromycin
ECOFF	Epidemiological cut-off value
EUCAST	European Committee on Antimicrobial Susceptibility Testing
F	Test statistics
Fe	Iron
FIC/FICI	Fractional inhibitory concentration/fractional inhibitory concentration index
GM	Gentamicin
HLGR	High-level aminoglycoside resistance
I	Susceptible, increased exposure
IQR	Interquartile range
LMM	Linear mixed model
LVX	Levofloxacin
LZD	Linezolid
MALDI–TOF MS	Matrix-assisted laser desorption/ionization time-of-flight mass spectrometry
MBC	Minimum bactericidal concentration
MDR MHB	Multidrug resistance Mueller–Hinton broth
MH-F	Mueller–Hinton broth for fastidious organisms
MIC	Minimum inhibitory concentration
MIC_50_	Antimicrobial concentration at which 50% of the tested isolates are inhibited
MIC_90_	Antimicrobial concentration at which 90% of the tested isolates are inhibited
MLS_B_	Resistance to macrolides, lincosamides and streptogramin B
MS_B_	Resistance to macrolides and streptogramin B
MXF	Moxifloxacin
NOR	Norfloxacin
OX	Oxacillin
P	Penicillin
*p*	Statistical significance
Q	Quartile
R	Resistant
RIF	Rifampicin
S	Susceptible, standard dosing
SXT	Trimethoprim/sulfamethoxazole
T	Tetracycline
TEC	Teicoplanin
TGC	Tigecycline
TOB	Tobramycin
WHO	World Health Organization
VA	Vancomycin
Zn	Zinc

## Appendix A

**Table A1:** The results of the post hoc tests (planned comparisons) in the analysis of the effect of species and antibiotic on antimicrobial activity.

Comparison	*p*
pyo: GM vs. AN	<0.0001
pyo: GM vs. TOB	0.0133
pyo: AN vs. TOB	<0.0001
aga: GM vs. AN	<0.0001
aga: GM vs. TOB	0.4744
aga: AN vs. TOB	<0.0001
pne: GM vs. AN	<0.0001
pne: GM vs. TOB	0.0289
pne: AN vs. TOB	0.0001
GM: pyo vs. aga	<0.0001
GM: pyo vs. pne	<0.0001
GM: aga vs. pne	0.0434
AN: pyo vs. aga	<0.0001
AN: pyo vs. pne	0.0561
AN: aga vs. pne	0.0229
TOB: pyo vs. aga	<0.0001
TOB: pyo vs. pne	<0.0001
TOB: aga vs. pne	0.2658
BTJ: pyo vs. aga	0.0286
BTJ: pyo vs. pne	0.7962
BTJ: aga vs. pne	0.3804
pyo: GM vs. BTJ	<0.0001
pyo: AN vs. BTJ	<0.0001
pyo: TOB vs. BTJ	0.0023
aga: GM vs. BTJ	0.7967
aga: AN vs. BTJ	<0.0001
aga: TOB vs. BTJ	0.0085
pne: GM vs. BTJ	1.0000
pne: AN vs. BTJ	<0.0001
pne: TOB vs. BTJ	0.0189

pyo—*S. pyogenes*; aga—*S. agalactiae*; pne—*S. pneumoniae*; GM—gentamicin; AN—amikacin; TOB—tobramycin; BTJ—β-thujaplicin; *p*—statistical significance.
