# Supplementary material for: Anti-Streptococcal Activity of β-Thujaplicin and Its Effect on Natural Low-Level Resistance of Streptococcus Species to Aminoglycoside Antibiotics
Source: Pathogens. 2026 Sep 15;15(9):978. doi: 10.3390/pathogens15090978 (PMC13610000; doi:10.3390/pathogens15090978)
Supplement: Supplementary file 1 [file pathogens-15-00978-s001.zip › pathogens-4461197-supplementary.pdf]

Table S1: MIC values of DMSO (BTJ solvent).

species	strain no.	MIC [%]	species	strain no.	MIC [%]	species	strain no.	MIC [%]
		DMSO			DMSO			DMSO
Streptococcus pyogenes	9ST	12,5	*Streptococcus agalactiae*	10ST	25	*Streptococcus pneumoniae*	17ST	25
	14ST	25		11ST	25		21ST	25
	18ST	25		12ST	25		22ST	12,5
	26ST	25		13ST	12,5		23ST	25
	27ST	25		15ST	25		24ST	25
	28ST	25		19ST	25		25ST	12,5
	29ST	25		20ST	25		35ST	25
	30ST	12,5		51ST	25		42ST	12,5
	31ST	25		55ST	25		46ST	25
	50ST	25		56ST	12,5		47ST	25
	ATCC 19615	25		ATCC 12386	25		ATCC 49619	12,5
	range	12,5 – 25		range	12,5 – 25		range	12,5 – 25

Table S2: MBC/MIC ratios for antibiotics and BTJ in the tested strains.

(a) - *S. pyogenes* , (b) - *S. agalactiae* , (c) *S. pneumoniae* , I - first measurement, II - second measurement)

(a) MBC I/MIC I

S. pyogenes												
strain no.	GM			AN			TOB			BTJ		
	MBC	MIC	MBC/MIC	MBC	MIC	MBC/MIC	MBC	MIC	MBC/MIC	MBC	MIC	MBC/MIC
ATCC19615	16	8	2,00	64	32	2,00	16	8	2,00	32	32	1,00
9 ST	32	16	2,00	256	128	2,00	64	64	1,00	32	32	1,00
14 ST	8	8	1,00	128	64	2,00	16	8	2,00	128	32	4,00
18 ST	16	8	2,00	128	64	2,00	32	16	2,00	32	16	2,00
26 ST	2	2	1,00	32	16	2,00	8	2	4,00	16	16	1,00
27 ST	8	4	2,00	32	32	1,00	16	8	2,00	32	32	1,00
28 ST	8	8	1,00	128	128	1,00	32	16	2,00	64	32	2,00
29 ST	8	8	1,00	128	64	2,00	32	16	2,00	64	32	2,00
30 ST	16	16	1,00	512	128	4,00	64	32	2,00	32	32	1,00
31 ST	8	2	4,00	128	32	4,00	8	2	4,00	128	32	4,00
50 ST	8	8	1,00	128	64	2,00	32	16	2,00	16	16	1,00

(b) MBC I/MIC I

S. agalactiae												
strain no	GM			AN			TOB			BTJ		
	MBC	MIC	MBC/MIC	MBC	MIC	MBC/MIC	MBC	MIC	MBC/MIC	MBC	MIC	MBC/MIC
ATCC12386	32	32	1,00	256	256	1,00	128	64	2,00	64	32	2,00
10 ST	128	128	1,00	512	512	1,00	256	128	2,00	512	128	4,00
11 ST	64	64	1,00	512	256	2,00	256	256	1,00	128	64	2,00
12 ST	64	64	1,00	256	256	1,00	128	128	1,00	32	16	2,00
13 ST	128	64	2,00	512	256	2,00	128	128	1,00	128	64	2,00
15 ST	32	32	1,00	256	128	2,00	32	32	1,00	256	32	8,00
19 ST	64	64	1,00	256	256	1,00	64	64	1,00	32	32	1,00
20 ST	64	32	2,00	128	128	1,00	32	32	1,00	512	64	8,00
51 ST	256	128	2,00	512	256	2,00	128	64	2,00	64	64	1,00
55 ST	128	64	2,00	256	256	1,00	128	128	1,00	32	32	1,00
56 ST	128	64	2,00	512	256	2,00	64	64	1,00	64	64	1,00

(c) MBC I/MIC I

S. pneumoniae												
strain no	GM			AN			TOB			BTJ		
	MBC	MIC	MBC/MIC	MBC	MIC	MBC/MIC	MBC	MIC	MBC/MIC	MBC	MIC	MBC/MIC
ATCC49619	8	8	1,00	16	8	2,00	16	16	1,00	64	32	2,00
17 ST	32	32	1,00	256	128	2,00	64	64	1,00	32	32	1,00
21 ST	32	32	1,00	128	128	1,00	64	64	1,00	32	32	1,00
22 ST	128	64	2,00	256	256	1,00	128	64	2,00	32	32	1,00
23 ST	32	32	1,00	128	64	2,00	32	32	1,00	32	16	2,00
24 ST	128	32	4,00	128	128	1,00	32	32	1,00	32	32	1,00
25 ST	16	16	1,00	128	128	1,00	64	32	2,00	32	32	1,00
35 ST	32	32	1,00	512	256	2,00	128	128	1,00	32	32	1,00
42 ST	64	32	2,00	256	128	2,00	64	64	1,00	64	32	2,00
46 ST	64	32	2,00	128	128	1,00	64	64	1,00	32	32	1,00
47 ST	32	32	1,00	256	128	2,00	128	64	2,00	32	32	1,00

MBC II/MIC II

S. pyogenes												
strain no.	GM			AN			TOB			BTJ		
	MBC	MIC	MBC/MIC	MBC	MIC	MBC/MIC	MBC	MIC	MBC/MIC	MBC	MIC	MBC/MIC
ATCC19615	16	8	2,00	64	64	1,00	32	16	2,00	16	16	1,00
9 ST	32	16	2,00	256	128	2,00	64	32	2,00	32	16	2,00
14 ST	8	8	1,00	128	64	2,00	16	8	2,00	128	16	8,00
18 ST	16	8	2,00	128	64	2,00	32	16	2,00	32	16	2,00
26 ST	2	2	1,00	64	32	2,00	8	2	4,00	16	16	1,00
27 ST	4	2	2,00	32	32	1,00	8	4	2,00	32	16	2,00
28 ST	8	8	1,00	128	128	1,00	32	16	2,00	32	16	2,00
29 ST	16	8	2,00	128	128	1,00	32	16	2,00	64	32	2,00
30 ST	16	16	1,00	512	128	4,00	64	32	2,00	32	16	2,00
31 ST	8	2	4,00	256	64	4,00	8	4	2,00	128	16	8,00
50 ST	8	8	1,00	128	64	2,00	16	16	1,00	16	16	1,00

MBC II/MIC II

S. agalactiae												
strain no	GM			AN			TOB			BTJ		
	MBC	MIC	MBC/MIC	MBC	MIC	MBC/MIC	MBC	MIC	MBC/MIC	MBC	MIC	MBC/MIC
ATCC12386	32	32	1,00	256	256	1,00	128	64	2,00	64	32	2,00
10 ST	128	128	1,00	512	512	1,00	256	256	1,00	512	64	8,00
11 ST	64	64	1,00	512	256	2,00	256	128	2,00	128	64	2,00
12 ST	64	64	1,00	512	512	1,00	128	64	2,00	16	8	2,00
13 ST	128	64	2,00	512	256	2,00	128	128	1,00	128	64	2,00
15 ST	32	16	2,00	128	64	2,00	32	32	1,00	256	32	8,00
19 ST	64	64	1,00	256	256	1,00	64	64	1,00	32	32	1,00
20 ST	32	32	1,00	256	256	1,00	32	32	1,00	512	64	8,00
51 ST	256	128	2,00	256	256	1,00	128	64	2,00	64	64	1,00
55 ST	64	64	1,00	256	256	1,00	128	128	1,00	64	32	2,00
56 ST	128	64	2,00	512	256	2,00	64	64	1,00	64	64	1,00

MBC II/MIC II

S. pneumoniae												
strain no	GM			AN			TOB			BTJ		
	MBC	MIC	MBC/MIC	MBC	MIC	MBC/MIC	MBC	MIC	MBC/MIC	MBC	MIC	MBC/MIC
ATCC49619	16	8	2,00	16	8	2,00	32	16	2,00	64	32	2,00
17 ST	32	32	1,00	256	128	2,00	64	64	1,00	32	16	2,00
21 ST	64	32	2,00	256	256	1,00	128	64	2,00	32	32	1,00
22 ST	128	64	2,00	512	256	2,00	128	128	1,00	32	32	1,00
23 ST	32	32	1,00	128	128	1,00	32	32	1,00	32	32	1,00
24 ST	128	64	2,00	256	256	1,00	32	32	1,00	32	32	1,00
25 ST	64	32	2,00	256	256	1,00	64	32	2,00	16	16	1,00
35 ST	32	32	1,00	512	256	2,00	128	128	1,00	32	32	1,00
42 ST	32	32	1,00	128	128	1,00	128	64	2,00	64	32	2,00
46 ST	64	32	2,00	128	128	1,00	64	64	1,00	32	32	1,00
47 ST	32	32	1,00	128	128	1,00	128	64	2,00	64	32	2,00
